# Body mass index and penile cancer incidence: results from a Norwegian cohort study of 829,081 men

**DOI:** 10.1186/s12894-024-01636-z

**Published:** 2024-11-27

**Authors:** Dagfinn Aune, Marie Nordsletten, Tor Åge Myklebust, Trude Eid Robsahm, Bjørn Steen Skålhegg, Tom Mala, Sheraz Yaqub, Usman Saeed

**Affiliations:** 1 Department of Research, Cancer Registry of Norway, Norwegian Institute of Public Health, Oslo, Norway; 2 Department of Nutrition, Oslo New University College, Oslo, Norway; 3 Department of Epidemiology and Biostatistics, School of Public Health, Imperial College London, White City Campus, 90 Wood Lane, London, W12 0BZ UK; 4 Department of Gastrointestinal and Paediatric Surgery, Oslo University Hospital, Oslo, Norway; 5 Department of Registration, Cancer Registry of Norway, Norwegian Institute of Public Health, Oslo, Norway; 6 Department of Research and Innovation, Møre and Romsdal Hospital Trust, Ålesund, Norway; 7 Division for Molecular Nutrition, Institute of Basic Medical Sciences, University of Oslo, Oslo, Norway; 8 Institute for Clinical Medicine, University of Oslo, Oslo, Norway

**Keywords:** Body mass index, penile cancer, cohort

## Abstract

**Background:**

A few previous studies have suggested a possible association between adiposity and increased risk of penile cancer, however, the evidence is to date limited for this rare cancer. We investigated the association between body mass index (BMI) and penile cancer risk in a large Norwegian cohort.

**Methods:**

The analyses included 829,081 men aged 16–75 years at baseline in 1963–1975. Multivariable Cox regression analyses were used to estimate hazard ratios (HRs) and 95% confidence intervals (CIs) for the associations between BMI and penile cancer incidence.

**Results:**

A total of 725 incident penile cancer cases occurred during 25.6 million person-years of follow-up. Compared to men with BMI 18.5-<25, the HRs (95% CIs) of those with a BMI of 15-<18.5, 25-<30, and ≥ 30 were 0.45 (0.15–1.41), 1.14 (0.97–1.33) and 1.63 (1.20–2.22), respectively, and the HR was 1.26 (1.12–1.42) per 5 kg/m^2^ increase in BMI. When the obese category was further subdivided in grade 1 (BMI 30-<35) and grade 2 obesity (≥ 35), the respective HRs were 1.52 (1.10–2.10) and 3.28 (1.46–7.35, p_trend_<0.001). The positive association persisted in sensitivity analyses excluding the first 5 years of follow-up. The association between BMI in early adulthood and penile cancer risk was less precise (1.23, 0.91–1.65 per 5 kg/m^2^, *n* = 143 cases) and for BMI and early-onset penile cancer was null (1.03, 0.51–2.06 per 5 kg/m^2^, *n* = 27 cases).

**Conclusion:**

High BMI is associated with increased risk of penile cancer. Further studies are needed to investigate the potential underlying mechanisms.

## Introduction

Penile cancer is rare with an age-standardized incidence rate of 0.80 cases per 100,000 person-years globally in 2020 [1, 2], and accounts for 0.4% of all cancer cases in men [1]. Globally, there is a wide range in the incidence rates of penile cancer with the highest rates observed in South America, southern parts of Africa, southeast Asia and northern Europe, and the lowest rates in northern Africa and the Middle East, and there is a 10-fold variation between countries with the highest rates (e.g. Uganda, Brazil) compared to countries with the lowest rates (e.g. Korea, Japan, Israel, Kuwait) [2]. There is evidence that the incidence of penile cancer has increased over time in several countries [2–8], although this has not been universally observed [2, 9]. These observations suggest modifiable risk factors may be important for the development of penile cancer. Established or suspected risk factors include lack of childhood/adolescent circumcision [10, 11], poor genital hygiene [12, 13], genital warts [14], phimosis [12, 15], human papilloma virus (HPV) infection [10], and smoking [10, 13, 15–17]. However, little else is known with regard to its aetiology.

Although excess weight has been established as a risk factor for 12 cancer sites according to the World Cancer Research Fund [18], few studies have investigated the association between adiposity and risk of penile cancer. A previous case-control study suggested a strong positive association between increasing BMI and penile cancer risk with odds ratios (95% CIs) of 2.64 (1.81–3.86) and 3.24 (2.07–5.08) for the overweight and obese vs. the normal weight category, respectively, and 1.53 (1.29–1.81) per 5 kg/m^2^ increase in BMI [19]. A large Danish registry-based study reported a standardized incidence ratio of 2.5 for penile cancer among men with a hospital diagnosis of obesity [20], and similarly a Swedish study reported a standardized incidence ratio of nearly 4 for a group of “other male genital cancers” (mainly penile cancer cases) among men with a hospital diagnosis of obesity [21]. Recently, a large pooled analysis of 12 Swedish cohorts with 2.1 million men reported a HR of 3.07 (2.28–4.14) for penile cancer for men with obesity vs. normal weight, and a HR of 1.54 (1.38–1.73) per 5 kg/m^2^ [22]. However, we are not aware of other studies that have investigated the association between adiposity and penile cancer risk. Given the low incidence, very large cohort studies would be needed to study this cancer. To shed further light on the association between BMI and penile cancer, we investigated the association in a large cohort of Norwegian men who participated in a nationwide tuberculosis screening program.

## Methods

The current study utilized weight and height measurements from the Norwegian Tuberculosis Screening Program (NTSP) conducted between 1963 and 1975 [23]. Weight and height were measured by health professionals. Data on cancer diagnoses were obtained by linkage to the Cancer Registry of Norway (CRN) using the personal identification number assigned to all Norwegian citizens to identify cancer cases. The CRN data have a high quality and are complete [24]. Linkage to the National Population Register provided information on date of death and emigration.

### Study population

A total of 1,911,598 individuals (aged 7–99 years) participated in the NTSP survey. The current analysis excluded all women (*n* = 993,598), those aged < 16 years and > 75 years (*n* = 66,974), those with missing data on weight or height (*n* = 765), those with BMI < 15 or > 50 kg/m^2^ (*n* = 70), men with a short stature (< 161 cm) (*n* = 13,722), those diagnosed with cancer (except cutaneous basal cell carcinoma) at baseline or within the first year of follow-up or with uncertain cancer diagnosis (*n* = 6,738), and those with no follow-up time (*n* = 650). The current analysis included 829,081 men aged 16–75 years at baseline after all exclusions were made.

### Outcome and follow-up data

Cancer diagnoses were obtained by linkage to the CRN using the International Classification of Diseases version 10 (ICD-10) codes. Penile cancer cases were identified by ICD-10 code C60. Individuals were followed from the NTSP screening date until penile cancer diagnosis, 75 years of age, death, emigration, or the end of follow-up (December 31, 2018), whichever came first. Only primary penile cancer cases were included in the current analysis.

### Statistical methods

We used multivariable Cox proportional hazards regression models to estimate HRs (95% CIs) for the association between BMI and penile cancer, adjusting for age groups at the time of screening, and using age as the underlying time scale. The proportional hazards assumption was tested using Schoenfeld’s test, and there was no deviation (*p* = 0.35). We categorized BMI using standard cut-off points 15-<18.5, 18.5-<25, 25-<30, ≥ 30, and also subdivided the latter category into 30-<35, and ≥ 35, to assess the impact of more extreme levels of adiposity on penile cancer risk. A test for linear trend was conducted by replacing the BMI category with the median value within each category and entering this variable as a continuous variable in the models. We also analysed the association per 5 kg/m^2^ increase in BMI.

We conducted analyses excluding the first 5 years of follow-up to further take into account reverse causation. We conducted analyses stratified by median height (> 175.1 vs. ≤175 cm) and tested for interactions between BMI and median height by incorporating cross-product terms between BMI categories and dichotomized height (> 175.1 vs. ≤175 cm) in the models, and by using a Wald test to test for differences between height subgroups.

Further analyses were conducted among individuals aged 16–29 years at baseline to assess the impact of BMI in early adulthood, and we also analysed the association between BMI and early-onset (< 50 years age at diagnosis) penile cancer risk. The statistical analyses were conducted using Stata 16.1 (StataCorp, College Station, TX, USA). A two-sided *p* < 0.05 was considered statistically significant.

## Results

The cohort included 829,081 men aged 16–75 years at baseline and the characteristics of the participants are shown in Table 1. Men with higher BMI were older and had higher weight and slightly lower height than men with BMI in the normal range. The mean follow-up was 32.5 (SD 14.8) years and during a total follow-up of more than 25.6 million person-years a total of 725 incident penile cancer cases occurred.

**Table 1:** Characteristics of the participants in the Norwegian tuberculosis screening program by BMI category

	Body mass index					
	< 18.5	18.5-<25.0	25.0-<30.0	30.0-<35.0	≥ 35.0	All
Participants, n (%)	12,297 (1.5%)	494,357 (59.6%)	282,710 (34.1%)	36,563 (4.4%)	3154 (0.4%)	829,081
Age at baseline (years), mean (SD)	30.1 (18.9)	39.7 (16.8)	48.6 (14.0)	52.4 (13.5)	52.5 (14.0)	43.2 (16.5)
BMI, mean (SD)	17.8 (0.6)	22.5 (1.6)	26.9 (1.3)	31.1 (1.2)	37.2 (2.2)	24.4 (3.2)
Weight, mean (SD)	54.9 (4.9)	69.5 (6.9)	81.9 (6.9)	95.2 (7.7)	112.4 (10.5)	74.8 (10.6)
Height, mean (SD)	175.5 (7.0)	175.8 (6.4)	174.5 (6.2)	173.8 (6.1)	173.8 (6.2)	175.2 (6.3)

The multivariable HRs (95% CIs) after adjustment for age were 0.45 (0.15–1.41), 1.00 (reference category), 1.14 (0.97–1.33) and 1.63 (1.20–2.22) for a BMI of 15-<18.5, 18.5-<25 (ref.), 25-<30, and ≥ 30, respectively, and the HR per 5 kg/m^2^ increase was 1.26 (1.12–1.42). When further subdividing the obese category in grade 1 (BMI 30-<35) and grade 2 obesity (≥ 35), the respective HRs were 1.52 (1.10–2.10) and 3.28 (1.46–7.35), and there was a significant test for linear trend (*p* < 0.001) across BMI categories. These positive associations persisted when excluding the first 5 years of follow-up, and were slightly strengthened (HR = 3.45, 1.54–7.75 for grade 2 obesity) (Table 2). When the analyses were stratified by median height, there was a significant interaction (*p* = 0.0026), with a somewhat more pronounced association in those who were taller (≥ 175.1 cm) with HRs of 0.69 (0.78–1.23), 1.00, 0.98 (0.78–1.23), 1.43 (0.86–2.39), and 3.89 (1.23–12.1) for BMI categories of 15-<18.5, 18.5-<25 (ref.), 25-<30, 30-<35, ≥ 35, respectively, than for those who were shorter (≤ 175.0 cm), for whom the corresponding HRs were 0.31 (0.04–2.22), 1.00, 1.31 (1.05–1.63), 1.65 (1.08–2.53), and 2.95 (0.94–9.25).

**Table 2:** Hazard ratios (HRs) and 95% confidence intervals (95% CIs) for the association between body mass index categories and the risk of penile cancer

	Body mass index (kg/m^2^)							
	15-<18.5	18.5-<25.0	25.0-<30.0	30.0-<35.0	≥ 35.0	≥ 30.0	Per 5 kg/m^2^	p_trend_
Person-years (years)	460,608	16,621,366	7,673,248	822,266	62,815	885,081	25,640,303	
Participants (n)	12,297	494,357	282,710	36,563	3154	39,717	829,081	
Cases^3^ (n)	-	399	276	41	-	-	725	
HR (95% CI)^1,4^	0.45 (0.15–1.41)	1.00	1.14 (0.97–1.33)	1.52 (1.10–2.10)	3.28 (1.46–7.35)	1.63 (1.20–2.22)	1.26 (1.12–1.42)	< 0.001
Person-years (years)	459,800	16,580,949	7,640,565	817,484	62,476	879,960	25,561,274	
Participants (n)	12,113	486,487	277,133	35,733	3087	38,820	814,553	
Cases^3^ (n)	-	386	260	40	-	-	695	
HR (95% CI)^2,4^	0.46 (0.15–1.44)	1.00	1.12 (0.95–1.31)	1.56 (1.12–2.16)	3.45 (1.54–7.75)	1.68 (1.23–2.29)	1.26 (1.11–1.42)	< 0.001

^1^ Adjusted for age

^2^ Adjusted for age, excluding first 5 years of follow-up

^3^ Number of cases have been suppressed for some categories because of low numbers (< 5 cases) in one of them

^4^ HR, hazard ratio, CI, confidence interval

In the subset of participants aged 16–29 years at baseline, there was a total of 143 incident penile cancer cases and the HR on a continuous scale per 5 kg/m^2^ increment in BMI was 1.23 (0.91–1.65) (Table 3). The analysis of the association between BMI and early-onset penile cancer was limited by few cases (*n* = 27) and did not show a clear association with a HR of 1.03 (0.51–2.06) per 5 kg/m^2^ increment (Table 4).

**Table 3:** BMI at age 16–29 years and penile cancer risk

	Body mass index (kg/m^2^)					
	15-<18.5	18.5-<25.0	25.0-<30.0	≥ 30.0	Per 5 kg/m^2^	p_trend_
Person-years (years)	383,362	7,923,621	1,386,464	118,888	9,812,335	
Participants (n)	8399	173,817	31,219	2793	216,228	
Cases^2^ (n)	0	119	-	-	143	
HR (95% CI)^1,3^	-	1	0.90 (0.57–1.42)	1.03 (0.25–4.17)	1.23 (0.91–1.65)	0.84

^1^ Adjusted for age

^2^ Numbers have been suppressed for two of the categories because of low numbers (< 5 cases) in one of the categories

^3^ HR, hazard ratio, CI, confidence interval

**Table 4:** BMI and early-onset (age < 50 years) penile cancer

	Body mass index (kg/m^2^)					
	15-<18.5	18.5-<25.0	25.0-<30.0	≥ 30.0	Per 5 kg/m^2^	p_trend_
Person-years (years)	270,518	6,499,529	1,801,474	175,019	8,746,542	
Participants (n)	9,608	338,910	142,055	15,235	505,808	
Cases^2^ (n)	0	-	-	-	27	
HR (95% CI)^1,3^	-	1.00	0.64 (0.21–1.90)	1.64 (0.21–12.42)	1.03 (0.51–2.06)	0.50

^1^ Adjusted for age

^2^ Numbers have been suppressed because of low numbers (< 5 cases) in two of the categories

^3^ HR, hazard ratio, CI, confidence interval

## Discussion

In this large-scale Norwegian cohort study we found a positive association between obesity and risk of penile cancer, with a 63% increase in risk for obesity overall when compared to normal weight, and a 52% and a 228% increase in risk for grade 1 and grade 2 obesity, respectively. The observed association persisted in sensitivity analyses excluding the first 5 years of follow-up. The association between BMI in early adulthood showed a similar, but less precise association, however, the association between BMI and early-onset penile cancer was null, but these latter analyses were limited by a low number of cases.

To our knowledge, this is the second large-scale cohort study to investigate the association between BMI and penile cancer incidence. Our findings are consistent with the results of a previous case-control study which reported an odds ratio of 3.24 for penile cancer among men with obesity vs. normal weight [19], with registry-based cohort studies on hospital diagnoses of obesity from Denmark and Sweden which reported standardized incidence ratios of 2.5 to 4-fold, respectively (20;21), and with a recent large pooled analysis of 12 Swedish cohorts with 2.1 million men which reported a HR of 3.07 (2.28–4.14) for men with obesity vs. normal weight, and a HR of 1.54 (1.38–1.73) per 5 kg/m^2^ [22]. In the current analysis, we observed a slightly stronger association between BMI and penile cancer in tall men when compared to shorter men. The reason for this interaction may be because BMI better estimates fat mass for taller men than for shorter men, however, further studies are needed to clarify this observation.

The biological mechanism(s) that could explain this association has not been adequately explored. It has been hypothesized that obesity could contribute to penile cancer risk through impaired genital hygiene and self-examination, and buried penis with accumulation of smegma and phimosis [19]. A few studies have reported positive associations between type 2 diabetes and penile cancer [25, 26], which might suggest insulin resistance could play a role, given the strong association between adiposity and type 2 diabetes [27]. Diabetes has also been associated with increased risk of genital warts [28, 29), balanitis [30], balanoposthitis [31], and phimosis [31], conditions strongly associated with increase penile cancer risk [12, 14–16]. Lastly, there is some evidence inflammation may play a role in penile cancer development [32] and adiposity is known to be related to low-grade chronic inflammation [33].

Limitations of the current study include a lack of information on other important risk factors that could confound the observed associations including smoking and HPV infection. Of note, smoking is a strong risk factor for penile cancer with ORs of reported in the literature [10, 16], however, since smokers tend to have a lower BMI than non-smokers [34, 35], any confounding from smoking would most likely have led to an underestimation of the observed association between BMI and penile cancer risk. Circumcision is very unusual in the Norwegian population examined in this period, thus less likely to have biased the results. The follow-up was long and it is possible that the weight of the participants have changed since baseline. Due to lack of repeated BMI measures we were not able to take into account any changes in BMI over time. Part of the observed association could therefore potentially be due to weight gain over time after the baseline. Strengths of the study include (1) the large sample size, which allowed analyses of this rare cancer site with sufficient statistical power, (2) use of weight and height measured and registered by healthcare professionals, which avoids errors with regard to self-reporting or recall bias, (3) linkages to cancer and death registries that are complete, and (4) up to 50 years follow-up with minimal loss to follow-up. Given the relatively strong and dose-dependent associations observed and consistency of the findings with the few previous studies that have been published [19–21], further large-scale cohort studies with more detailed data on confounding factors are warranted to clarify this association further.

In conclusion, in this large cohort study obesity grade 1 and grade 2 was associated with a 52% and 228% increase in penile cancer risk, respectively, when compared to normal weight. Further studies with more detailed data on confounding factors are needed to clarify these results and to clarify the underlying mechanisms.

## Data Availability

The data analysed in the current study is are not publicly available, but require applications for data access and ethical approval.
